# Hypoxic Preconditioning Enhances Cellular Viability and Pro-angiogenic Paracrine Activity: The Roles of VEGF-A and SDF-1a in Rat Adipose Stem Cells

**DOI:** 10.3389/fcell.2020.580131

**Published:** 2020-11-23

**Authors:** Yang Zhao, Ming Zhang, Guo-liang Lu, Bao-xing Huang, Da-wei Wang, Yuan Shao, Mu-jun Lu

**Affiliations:** ^1^Department of Urology, Ruijin Hospital, School of Medicine, Shanghai Jiao Tong University, Shanghai, China; ^2^Department of Urology, Ruijin Hospital North, School of Medicine, Shanghai Jiao Tong University, Shanghai, China; ^3^Department of Urology, Renji Hospital, School of Medicine, Shanghai Jiao Tong University, Shanghai, China

**Keywords:** adipose stem cells, hypoxic preconditioning, tissue engineering, cellular viability, pro-angiogenesis

## Abstract

To achieve the full therapeutic potential of implanted adipose stem cells (ASCs) *in vivo*, it is crucial to improve the viability and pro-angiogenic properties of the stem cells. Here, we first simulated the conditions of ischemia and hypoxia using the *in vitro* oxygen-glucose deprivation (OGD) model and confirmed that hypoxic preconditioning of ASCs could provide improved protection against OGD and enhance ASC viability. Second, we assessed the effect of hypoxic preconditioning on pro-angiogenic potential of ASCs, with a particular focus on the role of vascular endothelial growth factor-A (VEGF-A) and stromal derived factor-1a (SDF-1a) paracrine activity in mediating angiogenesis. We found that the conditioned medium of ASCs (ASC^CM^) with hypoxic preconditioning enhanced angiogenesis by a series of angiogenesis assay models *in vivo* and *in vitro* through the upregulation of and a synergistic effect between VEGF-A and SDF-1a. Finally, to investigate the possible downstream mechanisms of VEGF/VEGFR2 and SDF-1a/CXCR4 axes-driven angiogenesis, we evaluated relevant protein kinases involved the signal transduction pathway of angiogenesis and showed that VEGF/VEGFR2 and SDF-1a/CXCR4 axes may synergistically promote angiogenesis by activating Akt. Collectively, our findings demonstrate that hypoxic preconditioning may constitute a promising strategy to enhance cellular viability and angiogenesis of transplanted ASCs, therein improving the success rate of stem cell-based therapies in tissue engineering.

## Introduction

Recent developments in stem cell-based therapy have received considerable attention and produced promising results for tissue repair and functional tissue reconstruction after trauma. In the early days of tissue engineering, stem cells were used to promote tissue repair via migration to the damage sites and subsequent differentiation into tissue-specific cells (Hanson et al., 2010). However, this approach limited the potential benefits of stem cell-based therapy, because the viability of the implanted cells was strongly affected by microenvironment conditions in the implantation area. An injured tissue constitutes a hypoxic and ischemic environment that damage the implanted stem cells, leading to their apoptosis and thus compromising their differentiation capacity in the early stages of transplantation (Shafiq et al., 2016). Therefore, the current approach of tissue engineering is to use stem cells to promote tissue repair via pro-angiogenic paracrine effects rather than via colonization and differentiation (Vizoso et al., 2017).

In the past few years, gene modification methods had been used to strengthen the original genotype. However, such approaches disrupt genome stability and can cause unpredictable gene mutations, which reduce their potential use in clinical applications on safety grounds (Guo, 2017). Nowadays, preconditioning (non-genetic methods), which improve the viability of and pro-angiogenic factor release by stem cells, attracts considerable attention and considered to be safe and efficient for clinical use (Paquet et al., 2015).

Hypoxic preconditioning, through exposure to sub-lethal hypoxia stress, improve survival of stem cells and increase their resistance to deleterious injury in harsh migration environment by prior activation of cell survival pathways (Yue et al., 2013). Although severely hypoxic or anaerobic environments can cause cell death, transient and moderate hypoxia induces cytoprotection (Haider and Ashraf, 2010; Herrmann et al., 2011). Multiple studies have shown that hypoxia induces the expression of hypoxia inducible factor-1a (HIF-1a), which increase the release of a set of pro-angiogenic factors, including vascular endothelial growth factor-A (VEGF-A) and stromal cell-derived factor 1a (SDF-1a). A variety of pro-angiogenic factors protect adipose-derived stem cells (ASCs) from lethal hypoxia and other injuries, thus increases stem cell survival and angiogenesis around ischemic regions (Rosova et al., 2008; Stubbs et al., 2012). Therefore, hypoxic preconditioning is a promising method for enhancing cell viability and angiogenesis.

ASCs provide several advantages including convenient extraction, multi-directional differentiation potential, and secretion of a variety of pro-angiogenic factors, compared with bone marrow mesenchymal stem cells (BMSCs) (Harasymiak-Krzyzanowska et al., 2013; Kingham et al., 2014). More importantly, increasing studies have shown that ASCs can promote rapid angiogenesis in defective tissues (Rubina et al., 2009; Kim et al., 2013) through various mechanisms that may include paracrine activity of numerous angiogenic cytokines secreted by ASCs, induction and differentiation of ASCs into vascular endothelial cells, and promotion neovascular endothelial cell stability. Our previous findings also confirmed that ASCs can induce differentiation into urothelial cells and secrete a variety of pro-angiogenic factors (Zhang et al., 2014). Furthermore, we had used ASCs to repair bladder defects in a rat augmentation model and observed improved morphological regeneration and functional restoration (Zhe et al., 2016; Wang et al., 2017).

Therefore, to achieve the full therapeutic potential of implanted stem cells in tissue repair, and to improve the outcome of stem cell-mediated therapy after trauma, we applied the strategy of hypoxic preconditioning not only to improve the conditions of stem cells, but also to promote the release of pro-angiogenic factors. To our knowledge, this is the first study to confirm that hypoxia generated through pre-exposure to an oxygen-glucose deprivation (OGD) environment improves the viability of rat ASCs and promotes angiogenesis *in vivo* and *in vitro* via HIF-1a-VEGF-A/SDF-1a signaling. The optimum degree and duration of hypoxia in ASCs was determined for improving the survival, reducing cell damage and apoptosis, and maintaining the stem cell characteristics of rat ASCs. Furthermore, we also verified the central role of the VEGF/VEGFR2 and SDF-1a/CXCR4 axes in angiogenesis driven by hypoxia and investigated the underlying mechanisms.

## Materials and Methods

### Animals

The animal study was approved by the animal experimental review committee and performed in accordance with the guidelines and policies of Shanghai Jiao Tong University School of Medicine(license number: HKDL[2016]149). A total of twenty animals (2–3-week-old Sprague Dawley (SD) rats), were used to generate primary rat ASCs and twenty-five female athymic nude mice weighing 15–20 g were used for the establishment of the Matrigel plug angiogenesis assay *in vivo*.

### Isolation and Culture of Primary Rat ASCs

The abdominal subcutaneous adipose tissue was collected from 2–3 weeks old SD rats. The ASCs were isolated as described previously (Hsiao et al., 2012). Briefly, fresh adipose tissue was washed with phosphate-buffered saline (PBS) twice and sheared before digestion with 0.1% collagenase (type I; Sigma-Aldrich, St Louis, MO) followed by incubation at 37°C for 45 min. Then cells were centrifuged at 1000 rpm for 10 min and resuspended in complete DMEM and incubated at 37°C in an incubator.

### Hypoxia and OGD Model *in vitro*

ASCs were cultured under normoxic conditions in complete DMEM up to 80% confluency for all experiments. Two different methods were used to induce hypoxia at 2% and <0.1% O_2_ concentration. For the 2% O_2_ concentration, ASCs were cultured in a humidified incubator (Thermo) at 37°C, 2% O_2_. For a hypoxic environment with less than 0.1% O_2_, the hypoxia system Anaero Pouch–Anaero; (Mitsubishi Gas Chemical Company Inc., Japan) was used as previously described (Stubbs et al., 2012). The final indoor gas compositions were <0.1% O_2_ and 15% CO_2_. In order to mimic ischemia and anoxia *in vitro*, the OGD model was established using a previously published method (Chen et al., 2019). Similarly, in our study, the OGD exposure means that ASCs were cultured in serum- and glucose-free DMEM under hypoxic conditions (<0.1% O_2_) for 24 h.

To evaluate the effect of the degree and duration of hypoxia on ASCs exposed to OGD, the following experimental groups were established:

(a) Normoxic culture: ASCs were exposed to normoxic culture for 24 h followed by normoxic culture for 24 h.
(b) Normoxic preconditioning: ASCs were exposed to normoxic culture for 24 h followed by OGD exposure for 24 h.
(c) Hypoxic preconditioning at 2% O_2_: ASCs were exposed to hypoxic culture at 2% O_2_ for 24 h followed by OGD exposure for 24 h.
(d) Hypoxic preconditioning at <0.1% O_2_: ASCs were exposed to hypoxic culture at <0.1% O_2_ for 24 h followed by OGD exposure for 24 h.

### Assessment of Cellular Viability, Injury, Apoptosis, Stemness of ASCs

**FIGURE 1:**
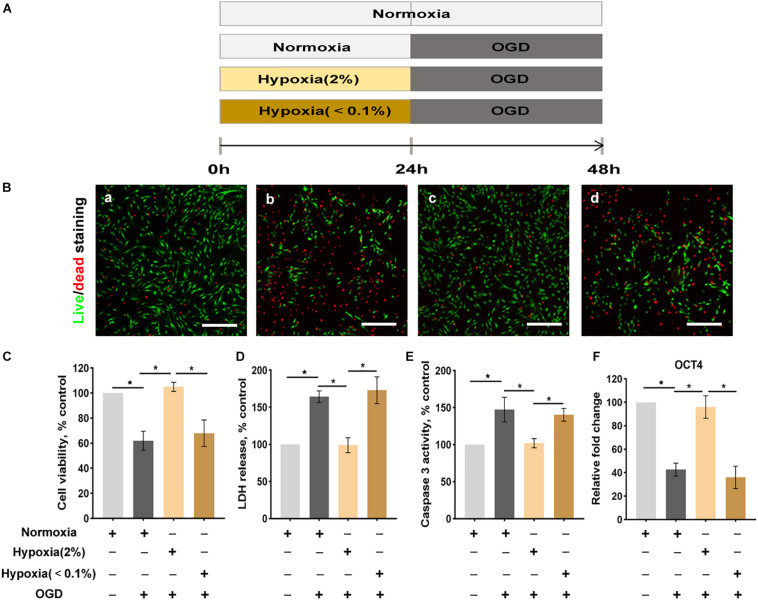
Hypoxia enhances the conditions of ASCs exposed to the oxygen-glucose deprivation (OGD) environment. **(A)** The OGD model *in vitro*. **(B)** The live/dead staining of ASCs: normoxia for 48 h **(a)**, normoxia 24 for h followed by OGD for 24 h **(b)**, hypoxia (2% O_2_) 24 h followed by OGD for 24 h **(c)**, hypoxia (< 0.1% O_2_) for 24 h followed by- OGD for 24 h **(d)**. Scale bar = 200 μm. **(C)** Hypoxia (2% O_2_) significantly improves cell viability (live/dead staining), **(D)** cellular injury (LDH release), **(E)** apoptosis (caspase 3 activity), and **(F)** stemness (Oct4 expression) of ASCs exposed to OGD. *n* = 6 per group; *denotes *P* < 0.05). LDH, lactate dehydrogenase.

The cellular viability was evaluated by Live/Dead^TM^ Viability/Cytotoxicity Kit (Thermo Fisher Scientific Inc.); the assay uses a two-color fluorescence system where living cells turn green and dead cells appear red. To better adhere to the wall, the 5 × 10^5^ ASCs were seeded in 35 mm culture plates and cultured for 24 h. Then these cells were exposed to preconditioning and OGD model separately (Figure 1A). After washing with PBS to remove serum esterase activity, ASCs seeded in plates were stained by 2 ml staining solution (an approximately 2 μM calcein AM, 4 μM EthD-1 solution and PBS) for 20 min at room temperature. The final images were observed and captured by a fluorescence microscope (Olympus). The quantity of live/dead cell was counted by random five fields. Statistical analysis of live dead assay was performed using Image J and is shown in Figure 1C.

Cellular injury was quantified by a LDH assay kit (Abcam). Briefly, a 60 μl LDH detection solution was prepared and added to supernatant at room temperature in dark for 30 min. The absorbance value was detected at 490 nm. Apoptosis was evaluated by measuring the caspase 3 activity. Briefly, the ASCs were collected, centrifuged and lysed on ice for 10 min and incubated with reaction buffer and DEVD-p-NA substrate for at 37°C 60–120 min. Caspase-3 activity was detected using an Caspase 3 Staining Kit (Abcam, ab39401) as described by the manufacturer’s instructions. The measurement results were showed on a microplate reader. The Oct4 (stemness marker) gene expression was detected by quantitative reverse transcription polymerase chain reaction (qRT-PCR) to evaluate the stemness of ASCs. OCT4: Forward: 5′- GCCCCCATTTCACCACACT -3′; Reverse: 5′-CCAGAGCAGTGACAGGAACA -3′.

### qRT-PCR

At the end of hypoxic (2% O_2_, <0.1% O_2_) or normoxic incubation periods of 6, 12, 24, or 48 h, total cellular RNA was extracted using TRIZOL. The complementary DNA (cDNA) was synthesized according to the manufacturer’s instructions. The qPCR was then conducted with Taqman^®^ technology using primers for the following genes: vascular endothelial growth factor-A (*VEGF-A*), vascular endothelial growth factor receptor 2 (*VEGFR2*), hepatocyte growth factor (*HGF*), stromal-derived factor-1a (*SDF-1a*), C-X-C chemokine receptor type 4 (*CXCR4*), basic fibroblast growth factor (*bFGF*), and beta-actin. Results were analyzed using the 2^–ΔΔct^ method. The design and examination of primers was professionally provided by Sangon Biotech company in shanghai.

VEGF-A Forward: 5′-AGGGCAGAATCATCACGAAGT-3′

VEGF-A Reverse: 5′-AGGGTCTCGATTGGATGGCA-3′

VEGFR2 Forward: 5′-TGATGGTGATGGTGCAGAAGGT-3′

VEGFR2 Reverse: 5′-AGAACCAGAGACCACATGGCT CG-3′

SDF-1a Forward: 5′-ATTCTCAACACTCCAAACTGTGC-3′

SDF-1a Reverse: 5′-ACTTTAGCTTCGGGTCAATGC-3′

CXCR4 Forward: 5′-ACTACACCGAGGAAATGGGCT-3′

CXCR4 Reverse: 5′-CCCACAATGCCAGTTAAGAAGA-3′

HGF Forward: 5′-CAGGAAAACTACTGTCGAAATC-3′

HGF Reverse: 5′-CTTCTGAACACTGTAAAGTTCTGC-3′

bFGF Forward: 5′-GATGCCGCTGGCAGCCATTGCCA-3′

bFGF Reverse: 5′-ATGGCTGCCAGCGGCATCCAAGTC-3′.

### Western Blot

ASCs were incubated in DMEM in 100 mm dishes under normoxia or hypoxia (2% O_2_, < 0.1% O_2_) for 24 h. The cells were then washed with cold PBS and lysed with RIPA buffer (10 mM Tris, pH 7.4, 150 mM NaCl, 1 mM EGTA, 0.1% SDS, 1 mM NaF, 1 mM Na_3_VO_4_, 1 mM phenylmethylsulfonyl fluoride, 1 mg/ml aprotinin, 1 mg/ml leupeptin). The protein concentration of each sample was determined using a BCA protein assay kit. The protein concentration of the lysates was quantified using a BCA assay Kit. Overall, 100mg protein was separated by electrophoresis and was transferred onto a Hybond nitrocellulose membrane. The membrane was then blocked with 5% nonfat milk and hybridized overnight at 4 °C with primary antibodies against HIF-1a (Santa Cruz; 1:200), VEGFR2 (Abcam; 1:500), CXCR4 (Abcam; 1:500), and b-actin (Sigma; 1:1000). Proteins were detected using enhanced chemiluminescence with horseradish peroxidase-conjugated secondary antibodies.

### Enzyme-Linked Immunosorbent Assay (ELISA)

The culture supernatant of ASCs from the NPC or HPC (2% O_2_) group was collected and used as the ASC-conditioned medium (ASC^CM^). Secreted VEGF-A and SDF-1a in the ASC^CM^ were quantified by a Quantikine ELISA kit (R&D systems and Wuhan Fine Biotech) according to the instructions. The protein concentrations of the lysates were quantified using a BCA assay Kit Absorbance was measured at 450 nm.

### Collection of ASC^CM^ and Growth Factor Pull-Down Assay

The ASC^CM^ was collected and centrifuged at 300 × *g* for 5 min to remove cellular debris and filtered through a 0.2 μm filter. To clarify the contribution of SDF-1a and VEGF-A in HPC-ASC^CM^-induced angiogenesis, the HPC-ASC^CM^ was incubated with neutralizing antibodies against VEGF-A (R&D, 25 μg/mL) and/or SDF-1a (Abcam, 10 μg/mL) and was continuously stirred at 4 °C for 2 h to allow the resin to bind to the neutralizing antibodies. Then, neutralizing antibody-bound SDF-1a and VEGF in HPC-ASC^CM^ were separated by centrifugation at 2000 rpm for 5 min. The supernatant was frozen at −80°C for follow-up experiments.

Experimental groups included: (1) NPC-ASC^CM^, (2) HPC-ASC^CM^, (3) HPC-ASC^CM^ with VEGF-A antibody, (4) HPC-ASC^CM^ with SDF-1a antibody, and (5) HPC-ASC^CM^ with VEGF-A and SDF-1a antibody.

### Wound Healing Assay *in vitro*

Wound healing assay *in vitro* was evaluated by cell migration. In brief, the human umbilical vein endothelial cells (HUVECs) were seeded and cultured in 6-well culture plates and incubated until they reach 90% confluence. At the center of each dish, the tip of the pipette was used to form a rectangular cell-free area. Then, cells were washed three times with PBS to remove original medium and floating cells. The ASC^CM^ of all groups was separately added to the dish followed by a 24-hour incubation. The representative images were captured under a microscope (Olympus). Wound closure was measured by the distance covered by the migrated cells.

### *In vitro* Angiogenesis Assay

Cultured HUVECs (1 × 10^5^ cells per well) were seeded on 24-well plates coated with Matrigel matrix (BD Biosciences). The 2 ml ASC^CM^ of all groups was separately added to the corresponding well and was incubated with the HUVECs at 37°C in an incubator. Tube formation was measured at 24 h. Each culture well was photographed using a Nikon TE-2000 camera (Japan). The number of tubes, junctions, branches and total tube length were quantified in each group by using the Image J plugin “Angiogenesis analyze” for Image J software.

### *In vivo* Matrigel Plug Angiogenesis Assay

Angiogenesis was assayed by growth of blood vessels from subcutaneous tissue into a solid gel of basement membrane containing the test sample (Malinda, 2009). Mixtures of 100 μl ASC^CM^ and 400 μl Matrigel matrix (BD Biosciences) were performed according to the manufacturer’s instructions. Matrigel mixture (500 μL) were subcutaneously injected into a flank of female athymic nude mice (one injection site per mouse) using a syringe with a 24G one-inch needle. The 25 nude mice were randomly divided into 5 groups, each had 5 mice. All equipment and reagents were chilled on ice prior to injection. After inoculation for 7 days, mice were euthanized and Matrigel plug was excised and fixed in 4% formaldehyde. Matrigel plugs were used by H&E staining, immunohistochemistry for CD31 and western blot for growth of blood vessels.

### HE Staining, Immunohistochemistry, and Western Blot of Angiogenesis in Matrigel Plug

The excised Matrigel plugs were rinsed with PBS and fixed in 10% neutral-buffered formalin, dehydrated in graded alcohols, and then embedded in paraffin in an optimum orientation to capture the entire BD Matrigel plug within each section. The sections (5 μm) were cut and then stained with HE for tissue morphology and stained immunohistochemically for CD31 (Abcam, ab28364, 1:50 dilution) to identify blood vessels. Sections with CD31 staining were analyzed using a Nikon TE-2000 camera (Japan). To minimize bias, the number of CD31 positive vessels were quantified in the whole tissue section of Matrigel plug by using Image J software. Unbiased stereological analyses were used to estimate % the area of the newly formed blood vessels/total area according to the Cavalieri principle (Howard and Reed, 1998). Morphometric counts need to be made independently by two observers blinded to treatment and the counts compared to ascertain that no more than 10% difference existed between counts from the two observers on the same tissue sections.

In order to remove the pollutants that might affect the stability of protein, the precooled neutral buffer was used for simple washing. After washing, the tissue was frozen rapidly in liquid nitrogen to retain the structure and characteristics of the protein. Tissue samples were preserved on ice and homogenized immediately. Western blotting was performed according to the manufacturer’s instructions (Abcam. The primary antibodies (Cell Signaling Technology) used to detect phosphorylation of Akt (p-Akt), Akt, phosphorylation of p38 (p-p38), p38, phosphorylation of ERK (p-ERK), ERK, and GAPDH. Quantitative analysis of protein expression was performed using Image J software.

### Statistical Analysis

Statistical analyses were performed using GraphPad Prism v8.0 Software. Data are expressed as mean ± standard deviation (SD) of at least three independent experiments. Statistical comparisons were made using unpaired t-test or one-way ANOVA. The results were considered statistically significant when *P* < 0.05.

## Results

### Hypoxia Enhances the Viability of ASCs Exposed to OGD

The viability of ASCs was evaluated by a live/dead staining assay where dead cells, which lose membrane integrity, appear red and living cells appear green (Figure 1B). The viability of cells in the normoxic preconditioning group was significantly reduced. Hypoxic preconditioning at 2% O_2_ group exhibited significantly higher cell viability than that of normoxic preconditioning group. Similarly, the cellular injury, as assessed by an LDH release assay, was also significantly lower in ASCs of the hypoxic preconditioning at 2% O_2_ group than in those of the normoxic preconditioning (Figure 1D). The caspase 3 activity assay showed that ASCs with hypoxic preconditioning(at 2% O_2_) were protected from OGD-induced cellular apoptosis (Figure 1E). In addition, the cell stemness, as assessed by Oct4 (stemness marker), was significantly reduced in the OGD environment. ASCs with hypoxic preconditioning(at 2% O_2_) had significantly higher cell stemness when challenged with OGD compared with the ASCs with normoxic preconditioning (Figure 1F).

### Hypoxia Upregulates HIF-1a Expression in ASCs

**FIGURE 2:**
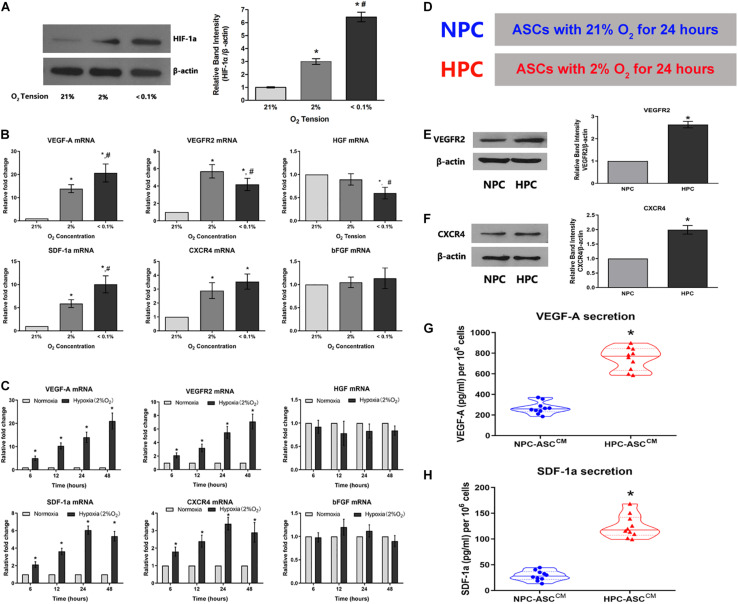
Hypoxia enhances the expression of the HIF-1a and the expression of its downstream pro-angiogenic genes in ASCs. **(A)** Hypoxia caused an O_2_-concentration-dependent increase in HIF-1a protein levels after 24 h (*n* = 5, * denotes *P* < 0.05 vs. the 21% O_2_ group, ^#^denotes *P* < 0.05 vs. the 2% O_2_ group). **(B,C)** The degree and duration of hypoxia regulate mRNA expression of the pro-angiogenic genes (VEGF-A, VEGFR2, HGF, SDF-1a, CXCR4, and bFGF) in ASC (*n* = 6, * denotes *P* < 0.05 vs. the 21% O_2_ group; ^#^denotes *P* < 0.05 vs. the 2% O_2_ group). **(D)** Normoxic preconditioning (NPC) indicates treatment of ASCs with 21% O_2_ for 24 h; hypoxic preconditioning (HPC) indicates treatment of ASCs with 2% O_2_ for 24 h. **(E,F)** Representative western blots of VEGFR2 and CXCR4 in ASCs with HPC and NPC. Quantification of VEGFR2 and CXCR4 protein levels (normalized to β-actin protein levels) was expressed as relative ratios (*n* = 5, * denotes *P* < 0.05 vs. NPC group). **(G,H)** HPC increases VEGF-A and SDF-1a secretion in the conditioned medium from ASCs (ASC^CM^) compared to the NPC (*n* = 10, *denotes *P* < 0.05 vs. NPC-ASC^CM^).

Given the fundamental role in the cellular response to hypoxia, we examined HIF-1a expression in ASCs subjected the varying oxygen levels (Figure 2A). As expected, HIF-1a expression in ASCs increased with decreasing oxygen concentration under 21%, 2%, and < 0.1% O_2_ after a 24 h preconditioning. ASCs maintained under lower oxygen levels (2% and < 0.1% O_2_) showed three to six-fold higher levels of HIF-1a expression (3.11 ± 0.22, 6.34 ± 0.39, respectively; *P* < 0.05). Further, there was a significant difference in HIF-1a expression between the 2% and < 0.1% oxygen preconditioning groups (3.11 ± 0.22, 6.34 ± 0.39, respectively; *P* < 0.05). These data demonstrate the success of the hypoxic preconditioning method.

### The Degree and Duration of Hypoxia Exposure Regulate mRNA Levels of the Pro-angiogenic Genes

To verify whether oxygen concentration affect mRNA levels of relevant pro-angiogenic factors, ASCs were respectively preconditioned in the environments with 21%, 2%, and less than 0.1% oxygen concentration for 24 h (Figure 2B). The mRNA levels of VEGF-A, VEGFR2, SDF-1a and CXCR4 were significantly up-regulated in 2% O_2_ group compared with that in the 21% O_2_ group (VEGF-A: 13.9 ± 1.7 fold, VEGFR2: 5.7 ± 0.8 fold, SDF-1a: 5.9 ± 0.8 fold, and CXCR4: 2.9 ± 0.6 fold); VEGF-A and SDF-1a levels were further increased as the oxygen concentration declined to less than 0.1% [VEGF-A: 21% O_2_ (1 fold), 2% O_2_ (13.9 ± 1.7 fold) and 0.1% O_2_ (20.7 ± 3.9 fold); SDF-1a: 21% O_2_ (1 fold), 2% O_2_ (5.9 ± 0.8 fold) and 0.1% O_2_ (10.0 ± 1.8 fold)]. However, the mRNA levels of bFGF were almost unaffected by oxygen concentration [VEGF-A: 21% O_2_ (1 fold), 2% O_2_ (1.0 ± 0.1 fold) and 0.1% O_2_ (1.1 ± 0.2 fold)]. In addition, HGF expression was not statistically different between the 2% and 21% O_2_ groups(1 fold and 0.9 ± 0.1 fold, respectively), but the levels decreased as the oxygen concentration dropped to below 0.1%(0.6 ± 0.1 fold). Thus, 2% O_2_ concentration was selected as the optimal condition for hypoxic preconditioning and used in the follow-up experiments.

ASCs were exposed to hypoxia (2% O_2_) for durations of 6, 12, 24, or 48 h to determine whether duration of hypoxia affects relevant pro-angiogenic mRNA expression (Figure 2C). The mRNA levels of VEGF-A were obviously increased with the duration: 6 h (5.1 ± 0.9 fold), 12 h (10.3 ± 1.3 fold), 24 h (14.3 ± 2.4 fold), and 48 h (21.1 ± 3.5 fold). VEGFR2 showed a similar trend, wherein the mRNA level gradually increased with time: 6 h (2.1 ± 0.3 fold), 12 h (3.2 ± 0.5 fold), 24 h (5.5 ± 0.8 fold), and 48 h (7.1 ± 1.1 fold). SDF-1a and CXCR4 mRNA levels were increased gradually and significantly with time: 6 h (2.1 ± 0.2 fold and 1.8 ± 0.3 fold, respectively), 12 h (3.6 ± 0.4 fold and 2.4 ± 0.3 fold, respectively), 24 h (6.1 ± 0.5 fold and 3.4 ± 0.4 fold, respectively), and 48 h (5.4 ± 0.5 fold, 2.9 ± 0.6 fold, respectively). However, the mRNA levels of HGF and bFGF were not influenced by the duration of hypoxia exposure. Thus, 24 h was determined as the optimum duration for the subsequent experiments.

In summary, we found that 2% O_2_ and 24 h as the optimum precondition was used to upregulate mRNA levels of the pro-angiogenic genes in the maximum extent. ASC culture at 21% O_2_ for 24 h (hereafter, NPC) and ASC culture at 2% O_2_ for 24 h (hereafter, HPC) were separately set up as control group and experimental group used in subsequent experiments (Figure 2D).

### HPC Increases VEGFR2 and CXCR4 Protein Levels and the Secretion of VEGF-A and SDF-1a

To verify whether augmented VEGF-A, VEGFR2, SDF-1a, and CXCR4 mRNA levels were converted into an corresponding protein increase, western blot and ELISA were performed. The VEGFR2 protein expression in HPC-ASCs was approximately 2.5-fold higher than that in NPC-ASCs (Figure 2E, *P* < 0.05). Similarly, we found that the expression of CXCR4 protein was significantly more (∼2-fold) in HPC-ASCs than in NPC-ASCs (Figure 2F, *P* < 0.05). The ASC^CM^ exposed to NPC or HPC was assessed for the secretion VEGF-A and SDF-1a by ELISA. The secretion of VEGF-A was significantly elevated (∼3-fold) in HPC-ASC^CM^ compared with that in NPC-ASC^CM^ (267.9 ± 58.1 vs. 748.8 ± 109.4 pg/ml, *P* < 0.05, 10^6^ cells; Figure 2G). SDF-1a secretion was significantly higher in HPC-ASC^CM^ than in NPC-ASC^CM^ (28.8 ± 9.7 vs. 124.3 ± 22.2 pg/ml, *P* < 0.05, per 10^6^ cells; Figure 2H).

### HPC-ASC^CM^ Promoted Angiogenesis by Wound Healing Assay *in vitro*

**FIGURE 3:**
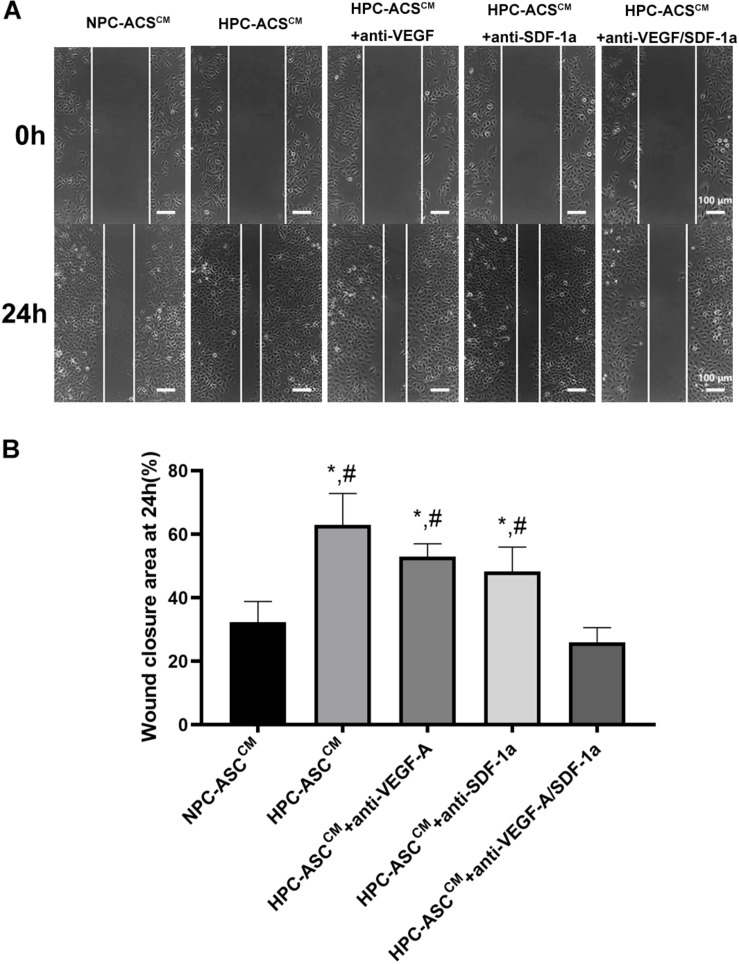
Wound healing assay *in vitro*. **(A)** The representative images of the HUVECs from each group at 0 and 24 h after scratching with a pipette head. Scale bar = 100 μm. **(B)** Quantitative analysis on wound closure as measured by the width covered by the migrated cells (*n* = 5, *denotes *P* < 0.05 vs. NPC-ASC^CM^; ^#^denotes *P* < 0.05 vs. HPC-ASC^CM^ + Anti-VEGF-A/SDF-1a).

The wound healing scratch assay was used to evaluate the migration and repair ability of HUVECs *in vitro*. The HUVECs were incubated in ASC^CM^ after scratching the 90% confluent cells with a pipette head (Figure 3A). Then wound closure area was measured by the width of the area covered by migrated cells at 0 and 24 h (Figure 3B). The wound closure area was significantly increased in the HPC-ASC^CM^ group (63.0 ± 9.8%, *P* < 0.05) and the HPC-ASC^CM^ group with removal of VEGF-A (53.0 ± 4.0%, *P* < 0.05) or SDF-1a (48.3 ± 7.6%, *P* < 0.05) alone compared with that in the NPC-ASC^CM^ group (32.3 ± 6.5%, *P* < 0.05) and the HPC-ASC^CM^ group with removal of VEGF-A and SDF-1a (26.0 ± 4.6%, *P* < 0.05). The removal of VEGF-A or SDF-1a alone did not result in a remarkable drop on the wound closure area in the HPC-ASC^CM^ group.

### HPC-ASC^CM^ Promoted Angiogenesis by Tube Formation Assay *in vitro*

**FIGURE 4:**
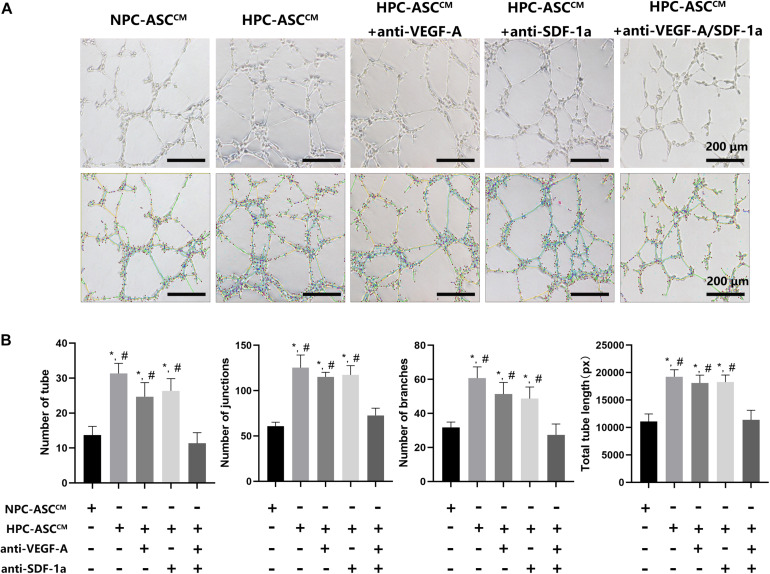
The tube formation assay *in vitro*. **(A)** Images of human umbilical vein endothelial cells after 24 h incubation with conditioned medium from ASC were analyzed by using the Image J plugin “Angiogenesis analyze” for Image J software. Scale bar = 200 μm. **(B)** The number of tubes, junctions, branches and total tube length were measured in each group. Image analysis was performed on three random fields per sample (*n* = 3 samples, *denotes *P* < 0.05 vs. NPC-ASC^CM^; ^#^denotes *P* < 0.05 vs. HPC-ASC^CM^ + anti-VEGF-A/SDF-1a).

Figure 4A shows the results of the tube-formation assay used to measure angiogenesis *in vitro*. The number of tubes, junctions, branches, and total tube length were considered indicators of angiogenesis (Figure 4B). The number of tube, junctions, branches and total tube length in the HPC-ASC^CM^ group (31.3 ± 2.9, 125.3 ± 14.0, 60.7 ± 6.7, 19242.8 ± 1262.4, respectively; *P* < 0.05) and the HPC-ASC^CM^ group with removal of VEGF-A (24.7 ± 4.0, 115.3 ± 5.2, 51.3 ± 6.8, 18089.0 ± 1451.0, respectively; *P* < 0.05) or SDF-1a (26.3 ± 3.5, 117.3 ± 10.2, 48.7 ± 6.8, 18257.3 ± 1302.9, respectively; *P* < 0.05) alone were all significantly higher than those in the NPC-ASC^CM^ group (13.6 ± 2.5, 60.7 ± 4.5, 31.7 ± 3.2, 11081.3 ± 1381.6, respectively; *P* < 0.05) and the HPC-ASC^CM^ group with removal of VEGF-A and SDF-1a (11.3 ± 3.1, 72.7 ± 8.1, 27.3 ± 6.4, 11391.7 ± 1734.6, respectively; *P* < 0.05). However, removal of VEGF-A or SDF-1a alone did not result in a remarkable drop in angiogenesis in the HPC-ASC^CM^ group (p > 0.05). There was no statistical difference in the angiogenesis between the NPC-ASC^CM^ group and the HPC-ASC^CM^ group with removal of VEGF-A and SDF-1a.

### HPC-ASC^CM^ Promoted Angiogenesis by Matrigel Plug Angiogenesis Assay *in vivo*

**FIGURE 5:**
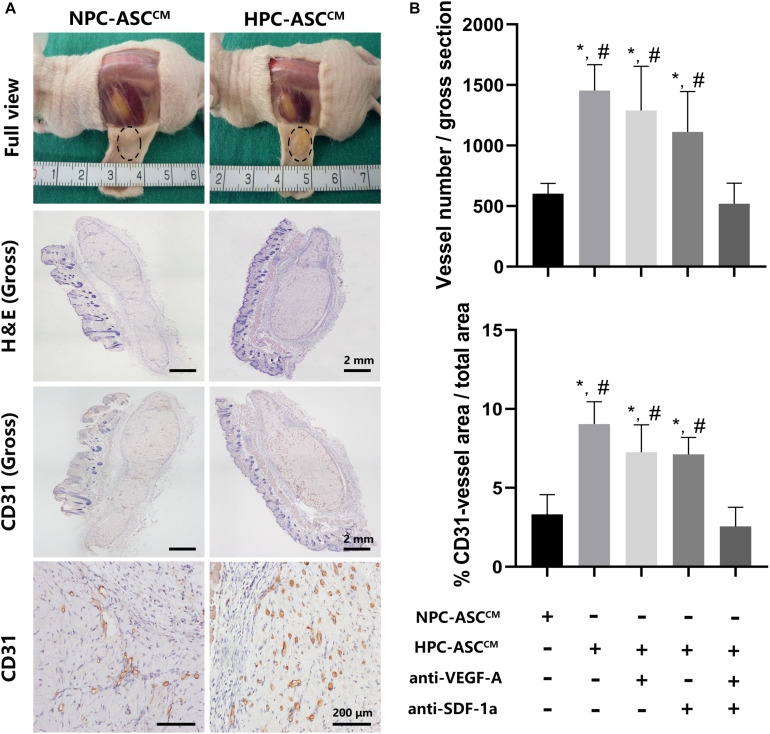
*In vivo* Matrigel plug angiogenesis assay. **(A)** Full view, H&E (Gross), CD31 (Gross, 200X) of the Matrigel plug one week after subcutaneous transplantation in nude mice. Gross (H&E, CD31): Scale bar = 2 mm. 200X (CD31): Scale bar = 200 μm. **(B)** Quantitative analysis on the number and area of CD31 positive vessels was measured. Image analysis was performed on three random fields per sample (*n* = 5 samples, *denotes *P* < 0.05 vs. NPC-ASC^CM^; ^#^denotes *P* < 0.05 vs. HPC-ASC^CM^ + anti-VEGF-A/SDF-1a).

In order to further verify the effect of ASC^CM^ on angiogenesis *in vivo*, Matrigel plug angiogenesis assay was conducted in nude mice. As shown in Figure 5, the *in vivo* results were highly consistent with the previous results *in vitro*. The number and area of the newly formed blood vessels was significantly increased in the HPC-ASC^CM^ group (1453.4 ± 214.4, 9.4 ± 1.4%, *P* < 0.05; respectively) and the HPC-ASC^CM^ group with removal of VEGF-A (1288.8 ± 365.8, 7.3 ± 1.7%, *P* < 0.05; respectively) or SDF-1a (1111.8 ± 334.4, 7.1 ± 1.1%, respectively; *P* < 0.05) alone compared with those in the NPC-ASC^CM^ group (603 ± 83.9, 3.3 ± 1.2%, respectively; *P* < 0.05) and the HPC- ASC^CM^ group with removal of VEGF-A and SDF-1a (518.4 ± 170.9, 2.6 ± 1.2%, respectively; *P* < 0.05). The removal of VEGF-A or SDF-1a alone did not result in an obvious drop in the number and area of vessels in the HPC-ASC^CM^ group. There was no statistical difference in the number and area of vessels between the NPC-ASC^CM^ group and the HPC- ASC^CM^ group with removal of VEGF-A and SDF-1a.

**FIGURE 6:**
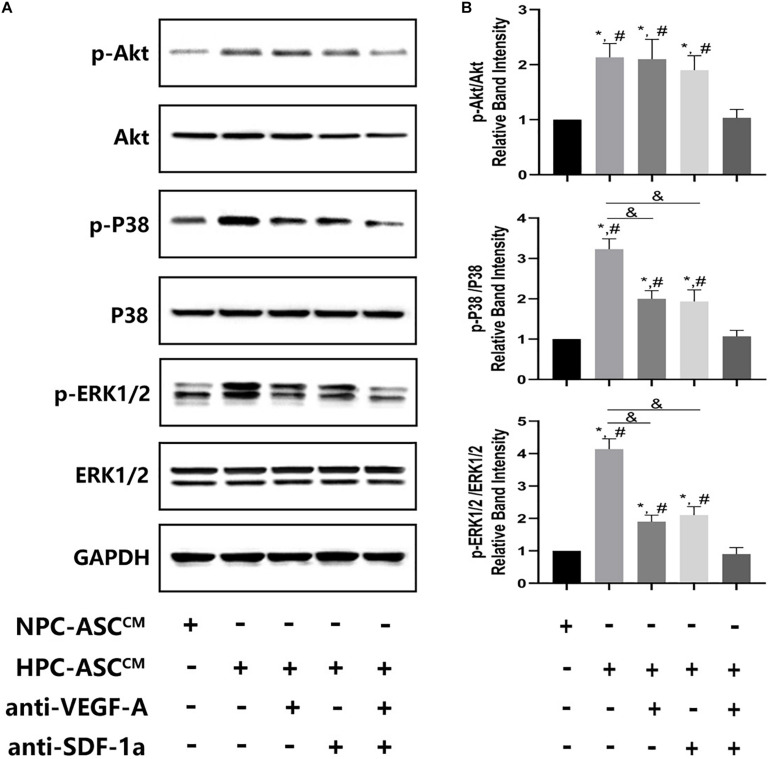
The expression of Akt, ERK, p38 at the protein level *in vivo* Matrigel plug. **(A)** Images of the western blots for Akt, ERK, and p38. **(B)** Quantitative analysis on protein expression of Akt, ERK, and p38 was measured. ^∗^denotes *P* < 0.05 vs. NPC-ASC^CM^; ^#^ denotes *P* < 0.05 vs. HPC-ASC^CM^ + anti-VEGF-A/SDF-1a; ^&^denotes *P* < 0.05.

### HPC-ASC^CM^ Promoted Angiogenesis by Akt Activation Through Synergetic Actions of VEGF-A and SDF-1a

To investigate the possible mechanism of synergism of VEGF-A and SDF-1a driven angiogenesis, we evaluated the phosphorylation of Akt, p38, ERK1/2, which are crucial proteins of various downstream pathways of angiogenesis, in the Matrigel plug *in vivo*. A significant increase in pAkt, p-p38, p-ERK1/2 expression was detected in the HPC-ASC^CM^ group and the HPC-ASC^CM^ group with removal of VEGF-A or SDF-1a alone compared with that in the NPC-ASC^CM^ group and the HPC-ASC^CM^ group with removal of VEGF-A and SDF-1a. However, removal of VEGF-A or SDF-1a alone did not result in a remarkable drop in pAkt expression in the HPC-ASC^CM^ group. Thus, VEGF-A and SDF-1a may synergistically promote angiogenesis by activating similar Akt signaling.

## Discussion

Stem cell-based therapy has a broad application prospect for the repair and functional reconstruction of tissue or organ defects in tissue engineering (Beisaw et al., 2020). Transplanting stem cells into the target area directly is a well-established approach. However, this method is associated with several problems including low cell retention and more cell damage, apoptosis, and even death. The quality of transplanted cells is critical for stem cell functionality, and the success of the therapy is closely related to the number of good-quality transplanted cells (Laflamme et al., 2007). In the past few years, researchers had tried several methods to improve cell survival and retention, for example through the modification of biological scaffolds (Kofidis et al., 2005), optimization of cellular carriers (Aguado et al., 2012), and gene modification (Khan et al., 2014). Another problem involves insufficient angiogenesis following stem cell transplantation. Preconditioning, which has better operability and feasibility in clinic, has been proven to promote cell viability and angiogenesis, both essential factors for successful cell therapy (Haider and Ashraf, 2010; Herrmann et al., 2011).

In the present study, hypoxia was used as the strategy for cell preconditioning. Hypoxic preconditioning provides a safe and clinically-applicable alternative and has been shown to induce a protective effect in many cell types (Mylotte et al., 2008; Rosova et al., 2008, Ciavarella et al., 2015). In general, transient and moderate hypoxia exposure is considered beneficial to cell survival, proliferation, and paracrine activity, but specific hypoxia strategies to improve the conditions of ASCs exposed to the OGD have rarely been reported. In addition, some studies focused only on the pro-angiogenic effect of hypoxic preconditioning (Chen et al., 2014; Han et al., 2016) and investigated mainly angiogenic factors, thus leaving other related mechanisms relatively underexplored.

The importance of the OGD model lies in its ability to closely mimic ischemia and anoxia *in vitro*. Cells are in a state of anoxia in the normal physiological environment of the body. Some think that there is almost no oxygen or nutrients available for the stem cells within 24 h after transplantation into the host (Xu et al., 2016). The frequently used methods of cobalt oxide induced hypoxia change the composition of culture medium, and it is difficult to control the degree and duration of hypoxia (Kang et al., 2014). In the last few years, however, hypoxia models have been greatly improved. The OGD model provides an ideal way to remove energy source, reduce oxygen supply, and showed good reproducibility (Ryou and Mallet, 2018). The OGD model lays a solid foundation for hypoxic preconditioning.

Generally, ASCs are cultured and used in normoxic incubators. However, the physiological “niches” for ASCs reside in subcutaneous fat tissue naturally, where the standard oxygen concentration is 1–2% (Cipolleschi et al., 1993). ASCs are particularly sensitive to the microenvironment because of powerful plasticity. The oxygen concentration is an important stimulus that plays a role in the intricate balance in many aspects of stem cell biology including their viability, proliferation, differentiation and migration (Valorani et al., 2012; Choi et al., 2014). In our OGD model *in vitro*, we show that hypoxia enhances the viability of ASCs exposed to OGD, which is consistent with the previous results (Choi et al., 2014; Bader et al., 2015). However, we have also found that the viability of ASCs in the normoxic preconditioning group has the similar result as the hypoxic preconditioning at <0.1% O_2_ group. The possible explanation is that the extreme hypoxia (<0.1% O_2_) may induces apoptosis or death of some cells that viability decrease (Follmar et al., 2006), while make the others stronger and more tolerant to OGD that viability improve (Stubbs et al., 2012).

In our model of hypoxic preconditioning, we found that the expression of HIF-1a protein was negatively correlated with the degree of hypoxia, *i.e.*, the protein was degraded under normoxia but stabilized and accumulated under hypoxia. It is well known that HIF-1a induces cellular responses by activating its downstream target genes, some of which enhance angiogenesis (Rios-Navarro et al., 2018). Our results are consistent with previous research (Liu et al., 2013, 2015), in which hypoxia was used to activate HIF-1a that lead to upregulation of downstream pro-angiogenic genes, such as VEGF-A, SDF-1a or other factors. It may involve a potential oxygen sensing pathway, which regulated a range of pro-angiogenic factors in ASC. Since its discovery, VEGF-A has been studied in various pro-angiogenesis fields of tissue engineering (Peach et al., 2018). Some researchers over-expressed VEGF-A by gene modification to achieve a continuous release of pro-angiogenic factors, which lead to a reduction in infarct size and an increase in angiogenesis (Hao et al., 2007; Deuse et al., 2009). In our study, VEGFR2 mRNA expression significantly increased after hypoxic preconditioning, in accordance with previous results suggesting that hypoxia regulates VEGFR2 mRNA expression in the bone marrow-derived cells (Okuyama et al., 2006). Furthermore, short-term exposure of ASCs to hypoxia was reported to result in an increase in stromal-derived factor-1 (SDF-1) and its receptor CXCR4 mRNA expression, which are involved in cell proliferation, survival, and angiogenesis (Liu et al., 2010). An increasing number of studies are now exploring the role of SDF-1 in the new angiogenesis model, indicating the central role of the SDF-1a/CXCR4 axis in angiogenesis for supporting tissue repair (Petit et al., 2007). Similar to VEGF-A, SDF-1a overexpression in BMSCs can improve cardiac function by increasing angiogenesis (Gong et al., 2019). However, the use of genetically modified cells brings inevitable risks in clinical application. Therefore, hypoxia (non-genetic methods), which could increase both VEGF-A and SDF-1a paracrine signaling from ASCs, may represent a safe and efficient method that is applicable in the clinic.

In our study, 2% O_2_ was selected as the optimal condition for hypoxic preconditioning and used to upregulate the pro-angiogenic mRNA levels. These results were closely related to previous findings that the physiological oxygen concentration of ASCs reside in subcutaneous fat tissue naturally is 1–2% (Cipolleschi et al., 1993). In our study, we found that mRNA levels of the pro-angiogenic genes of ASCs against the degree of hypoxia exposure seems to have a bell-shaped curve; That is to say, at a suitable degree of hypoxia exposure, pro-angiogenic function of ASCs is relatively most powerful. If above or below certain standard, these function will downregulate significantly. This result was consistent with other findings (Buizer et al., 2018) that exposing mesenchyma stem cell (MSCs) to 1–2% oxygen concentration lead to the most cell proliferation and expression of pro-angiogenic factor. This may indicate that 1%–2% oxygen stimulate vascular ingrowth the most. In addition, The bell-shaped curve also exists in duration of hypoxia exposure. Previous research reports (Bader et al., 2015) that MSCs were preconditioned by hypoxic incubation (1% O_2_) for 12, 24 or 36 h or under normoxic standard conditions. Here, the cell survival was higher and pro-angiogenic capacity was proved to be most efficient.

We also found that HPC could promote the paracrine angiogenesis both *in vitro* and *in vivo*. The previous studies have found that the transplantation of stem cells with hypoxic preconditioning could be conducive to functional recovery of damaged tissues (Bader et al., 2015). In this study, a definite evidence was provided by hypoxic ASC^CM^. Furthermore, we believe that VEGF-A and SDF-1a are major pro-angiogenic paracrine factors that regulate the enhanced angiogenesis of hypoxic ASC^CM^. When either VEGF-A or ANG was depleted by neutralizing antibodies from hypoxic ASC^CM^, there was no significant reduction in angiogenesis *in vitro* and *in vivo*. This might be explained by the possible notion that VEGF-A and SDF-1 have overlapping functions to a larger degree in the angiogenic process of hypoxic ASC^CM^. The removal of either VEGF-A or SDF-1 from hypoxic ASC^CM^ could be offset by the other one. However, if VEGF-A and SDF-1 are simultaneously absent, angiogenic capacity of hypoxic ASC^CM^ was significantly impaired, which suggests that pro-angiogenesis of hypoxic ASC^CM^ may be not depended on some kind of pro-angiogenic factor, but an assembly of core paracrine factors, which together make a central contribution to angiogenic properties. Moreover, both VEGF-A and SDF-1a paracrine actions are upregulated by upstream factor HIF-1a (Petit et al., 2007). These two factors can regulate each other’s expression. SDF-1 upregulates VEGF-A expression (Grunewald et al., 2006; Zisa et al., 2011) and vice versa. VEGF-A also increases the secretion of SDF-1 and CXCR4 (Liu et al., 2011). As previously reported, the above results might be explained by a synergistic interaction between VEGF-A and SDF-1a (Kryczek et al., 2005; Carr et al., 2006). These synergistic effects and compensatory mechanisms double guarantee the pro-angiogenesis effect of hypoxic ASC^CM^, which has not been studied in ASCs with the hypoxic preconditioning.

To study further the downstream signaling pathway of synergism of VEGF-A and SDF-1a driven angiogenesis induced by hypoxic ASC^CM^, we tested the expression of crucial protein kinases in the signal transduction pathway involved in angiogenesis. We found that deletion of either VEGF-A or SDF-1 could not lead to a significant reduction in pAkt expression; only simultaneous removal of VEGF-A and SDF-1a resulted in a remarkable drop, which was consistent with results obtained by the angiogenesis assay *in vitro* and *in vivo*. Thus, these data suggest that VEGF-A and SDF-1a may synergistically promote angiogenesis by activating Akt signaling in a similar manner. In the mouse ischemic limb model, VEGF-A induces a pro-angiogenesis effect through the activation of SDF-1–CXCR4 axis (Jin et al., 2006). In addition, VEGF-A and SDF-1a/CXCR4 axis also induce neo-angiogenesis synergistically in human ovarian cancers (Kryczek et al., 2005). Thus, the study of the angiogenesis contribution of VEGF-A/VEGFR2 and SDF-1a/CXCR4 signaling pathway is essential to understand and regulate the neo-angiogenesis during tissue regeneration.

The limitation of above study is lack of comparable result in stem cell survival and multilineage differentiation between transplanted NPC-ASCs and HPC-ASCs in animal model. In addition, the optimum degree and duration of hypoxia exposure is distinct in the different types of transplanted stem cells, which also increases the complexity of hypoxic preconditioning. Future studies will focus on evaluating effect of transplanted HPC-ASCs in the tissue repair and functional reconstruction *in vivo*, and promoting the clinical transformation of hypoxic preconditioning technology in tissue engineering.

In conclusion, hypoxia improves the conditions of ASCs exposed to OGD and significantly upregulates paracrine secretion of pro-angiogenic factors VEGF-A and SDF-1a. Using a series of angiogenesis assay models *in vivo* and *in vitro*, we provide robust evidence for pro-angiogenic paracrine activity of ASCs and show that hypoxic ASC^CM^ enhances angiogenesis through a synergistic effect between VEGF-A and SDF-1a. Furthermore, VEGF/VEGFR2 and SDF-1a/CXCR4 may synergistically promote angiogenesis by activating similar Akt signaling. Collectively, our work suggest that hypoxic preconditioning exerts the most promising approach for improving the conditions and pro-angiogenic activity of adipose stem cells in the tissue repair and functional reconstruction.

## Data Availability Statement

The original contributions presented in the study are included in the article/supplementary material, further inquiries can be directed to the corresponding authors.

## Ethics Statement

The animal study was reviewed and approved by Shanghai Jiao Tong University School of Medicine.

## Author Contributions

YZ, MZ, YS, and M-jL conceived and designed the experiments. YZ and MZ performed the experiments and wrote the first draft of the manuscript. G-lL, B-xH, and D-wW analyzed the data. All authors contributed to manuscript revision, read and approved the submitted version.

## Conflict of Interest

The authors declare that the research was conducted in the absence of any commercial or financial relationships that could be construed as a potential conflict of interest.

## References

[B1] Aguado B. A. Mulyasasmita W. Su J. Lampe K. J. Heilshorn S. C. (2012). Improving viability of stem cells during syringe needle flow through the design of hydrogel cell carriers. *Tissue Eng. Part A* 18 806–815. 10.1089/ten.tea.2011.0391 22011213 PMC3313609

[B2] Bader A. M. Klose K. Bieback K. Korinth D. Schneider M. Seifert M. (2015). Hypoxic preconditioning increases survival and pro-angiogenic capacity of human cord blood mesenchymal stromal cells in vitro. *PLoS One* 10:e0138477. 10.1371/journal.pone.0138477 26380983 PMC4575058

[B3] Beisaw A. Kuenne C. Gunther S. Dallmann J. Wu C. C. Bentsen M. (2020). AP-1 contributes to chromatin accessibility to promote sarcomere disassembly and cardiomyocyte protrusion during zebrafish heart regeneration. *Circ. Res.* 126 1760–1778. 10.1161/circresaha.119.316167 32312172 PMC7274905

[B4] Buizer A. T. Bulstra S. K. Veldhuizen A. G. Kuijer R. (2018). The balance between proliferation and transcription of angiogenic factors of mesenchymal stem cells in hypoxia. *Connect. Tissue Res.* 59 12–20. 10.1080/03008207.2017.1289189 28165799

[B5] Carr A. N. Howard B. W. Yang H. T. Eby-Wilkens E. Loos P. Varbanov A. (2006). Efficacy of systemic administration of SDF-1 in a model of vascular insufficiency: support for an endothelium-dependent mechanism. *Cardiovasc. Res.* 69 925–935. 10.1016/j.cardiores.2005.12.005 16409996

[B6] Chen L. Xu Y. Zhao J. Zhang Z. Yang R. Xie J. (2014). Conditioned medium from hypoxic bone marrow-derived mesenchymal stem cells enhances wound healing in mice. *PLoS One* 9:e96161. 10.1371/journal.pone.0096161 24781370 PMC4004560

[B7] Chen P. Zhang H. Zhang Q. Zhou W. Deng Y. Hu X. (2019). Basic fibroblast growth factor reduces permeability and apoptosis of human brain microvascular endothelial cells in response to oxygen and glucose deprivation followed by reoxygenation via the fibroblast growth factor receptor 1 (FGFR1)/ERK pathway. *Med. Sci. Monit.* 25 7191–7201. 10.12659/msm.918626 31551405 PMC6778414

[B8] Choi J. R. Pingguan-Murphy B. Wan Abas W. A. Noor Azmi M. A. Omar S. Z. Chua K. H. (2014). Impact of low oxygen tension on stemness, proliferation and differentiation potential of human adipose-derived stem cells. *Biochem. Biophys. Res. Commun.* 448 218–224. 10.1016/j.bbrc.2014.04.096 24785372

[B9] Ciavarella C. Fittipaldi S. Pedrini S. Vasuri F. Gallitto E. Freyrie A. (2015). In vitro alteration of physiological parameters do not hamper the growth of human multipotent vascular wall-mesenchymal stem cells. *Front. Cell Dev. Biol.* 3:36. 10.3389/fcell.2015.00036 26090364 PMC4455290

[B10] Cipolleschi M. G. Dello Sbarba P. Olivotto M. (1993). The role of hypoxia in the maintenance of hematopoietic stem cells. *Blood* 82 2031–2037. 10.1182/blood.v82.7.2031.bloodjournal8272031 8104535

[B11] Deuse T. Peter C. Fedak P. W. Doyle T. Reichenspurner H. Zimmermann W. H. (2009). Hepatocyte growth factor or vascular endothelial growth factor gene transfer maximizes mesenchymal stem cell-based myocardial salvage after acute myocardial infarction. *Circulation* 120(Suppl. 11) S247–S254. 19752375 10.1161/CIRCULATIONAHA.108.843680

[B12] Follmar K. E. Decroos F. C. Prichard H. L. Wang H. T. Erdmann D. Olbrich K. C. (2006). Effects of glutamine, glucose, and oxygen concentration on the metabolism and proliferation of rabbit adipose-derived stem cells. *Tissue Eng.* 12 3525–3533. 10.1089/ten.2006.12.3525 17518688

[B13] Gong X. H. Liu H. Wang S. J. Liang S. W. Wang G. G. (2019). Exosomes derived from SDF1-overexpressing mesenchymal stem cells inhibit ischemic myocardial cell apoptosis and promote cardiac endothelial microvascular regeneration in mice with myocardial infarction. *J. Cell Physiol.* 234 13878–13893. 10.1002/jcp.28070 30720220 PMC13483023

[B14] Grunewald M. I Avraham Y. Dor Bachar-Lustig E. Itin A. Jung S. Chimenti S. (2006). VEGF-induced adult neovascularization: recruitment, retention, and role of accessory cells. *Cell* 124 175–189. 10.1016/j.cell.2005.10.036 16413490

[B15] Guo Y. (2017). Role of HIF-1a in regulating autophagic cell survival during cerebral ischemia reperfusion in rats. *Oncotarget* 8 98482–98494. 10.18632/oncotarget.21445 29228704 PMC5716744

[B16] Haider H. Ashraf M. (2010). Preconditioning and stem cell survival. *J. Cardiovasc. Transl. Res.* 3 89–102. 10.1007/s12265-009-9161-2 20560023

[B17] Han K. H. Kim A. K. Kim M. H. Kim D. H. Go H. N. Kim D. I. (2016). Enhancement of angiogenic effects by hypoxia-preconditioned human umbilical cord-derived mesenchymal stem cells in a mouse model of hindlimb ischemia. *Cell Biol. Int.* 40 27–35. 10.1002/cbin.10519 26222206

[B18] Hanson S. E. Bentz M. L. Hematti P. (2010). Mesenchymal stem cell therapy for nonhealing cutaneous wounds. *Plast. Reconstr. Surg.* 125 510–516. 10.1097/prs.0b013e3181c722bb 20124836 PMC4076140

[B19] Hao X. Mansson-Broberg A. Grinnemo K. H. Siddiqui A. J. Dellgren G. Brodin L. A. (2007). Myocardial angiogenesis after plasmid or adenoviral VEGF-A(165) gene transfer in rat myocardial infarction model. *Cardiovasc. Res.* 73 481–487. 10.1016/j.cardiores.2006.10.011 17134685

[B20] Harasymiak-Krzyzanowska I. Niedojadlo A. Karwat J. Kotula L. Gil-Kulik P. Sawiuk M. (2013). Adipose tissue-derived stem cells show considerable promise for regenerative medicine applications. *Cell Mol. Biol. Lett.* 18 479–493. 23949841 10.2478/s11658-013-0101-4 PMC6275722

[B21] Herrmann J. L. Abarbanell A. M. Weil B. R. Manukyan M. C. Poynter J. A. Brewster B. J. (2011). Optimizing stem cell function for the treatment of ischemic heart disease. *J. Surg. Res.* 166 138–145. 10.1016/j.jss.2010.05.057 20828719 PMC3008759

[B22] Howard C. Reed M. (1998). *Unbiased Stereology. Three-Dimensional Measurement in Microscopy.* Milton Park: BIOS Scientific Publishers.

[B23] Hsiao S. T. Asgari A. Lokmic Z. Sinclair R. Dusting G. J. Lim S. Y. (2012). Comparative analysis of paracrine factor expression in human adult mesenchymal stem cells derived from bone marrow, adipose, and dermal tissue. *Stem Cells Dev.* 21 2189–2203. 10.1089/scd.2011.0674 22188562 PMC3411362

[B24] Jin D. K. Shido K. Kopp H. G. Petit I. Shmelkov S. V. Young L. M. (2006). Cytokine-mediated deployment of SDF-1 induces revascularization through recruitment of CXCR4+ hemangiocytes. *Nat. Med.* 12 557–567. 10.1038/nm1400 16648859 PMC2754288

[B25] Kang S. Kim S. M. Sung J. H. (2014). Cellular and molecular stimulation of adipose-derived stem cells under hypoxia. *Cell Biol. Int.* 38 553–562. 10.1002/cbin.10246 24446066

[B26] Khan M. Mohsin S. Toko H. Alkatib M. Nguyen J. Truffa S. (2014). Cardiac progenitor cells engineered with betaARKct have enhanced beta-adrenergic tolerance. *Mol. Ther.* 22 178–185. 10.1038/mt.2013.200 24002692 PMC3978798

[B27] Kim J. H. I Park S. Park Y. Jung Y. Kim S. H. Kim S. H. (2013). Therapeutic angiogenesis of three-dimensionally cultured adipose-derived stem cells in rat infarcted hearts. *Cytotherapy* 15 542–556. 10.1016/j.jcyt.2012.11.016 23352461

[B28] Kingham P. J. Kolar M. K. Novikova L. N. Novikov L. N. Wiberg M. (2014). Stimulating the neurotrophic and angiogenic properties of human adipose-derived stem cells enhances nerve repair. *Stem Cells Dev.* 23 741–754. 10.1089/scd.2013.0396 24124760

[B29] Kofidis T. Lebl D. R. Martinez E. C. Hoyt G. Tanaka M. Robbins R. C. (2005). Novel injectable bioartificial tissue facilitates targeted, less invasive, large-scale tissue restoration on the beating heart after myocardial injury. *Circulation* 112(Suppl. 9) I173–I177. 16159811 10.1161/CIRCULATIONAHA.104.526178

[B30] Kryczek I. Lange A. Mottram P. Alvarez X. Cheng P. Hogan M. (2005). CXCL12 and vascular endothelial growth factor synergistically induce neoangiogenesis in human ovarian cancers. *Cancer Res.* 65 465–472. 15695388

[B31] Laflamme M. A. Chen K. Y. Naumova A. V. Muskheli V. Fugate J. A. Dupras S. K. (2007). Cardiomyocytes derived from human embryonic stem cells in pro-survival factors enhance function of infarcted rat hearts. *Nat. Biotechnol.* 25 1015–1024. 10.1038/nbt1327 17721512

[B32] Liu G. Lu P. Li L. Jin H. He X. Mukaida N. (2011). Critical role of SDF-1alpha-induced progenitor cell recruitment and macrophage VEGF production in the experimental corneal neovascularization. *Mol. Vis.* 17 2129–2138. 21850188 PMC3156784

[B33] Liu H. Xue W. Ge G. Luo X. Li Y. Xiang H. (2010). Hypoxic preconditioning advances CXCR4 and CXCR7 expression by activating HIF-1alpha in MSCs. *Biochem. Biophys. Res. Commun.* 401 509–515. 10.1016/j.bbrc.2010.09.076 20869949

[B34] Liu J. Hao H. Xia L. Ti D. Huang H. Dong L. (2015). Hypoxia pretreatment of bone marrow mesenchymal stem cells facilitates angiogenesis by improving the function of endothelial cells in diabetic rats with lower ischemia. *PLoS One* 10:e0126715. 10.1371/journal.pone.0126715 25996677 PMC4440823

[B35] Liu L. Gao J. Yuan Y. Chang Q. Liao Y. Lu F. (2013). Hypoxia preconditioned human adipose derived mesenchymal stem cells enhance angiogenic potential via secretion of increased VEGF and bFGF. *Cell Biol. Int.* 37 551–560. 10.1002/cbin.10097 23505143

[B36] Malinda K. M. (2009). In vivo matrigel migration and angiogenesis assay. *Methods Mol. Biol.* 467 287–294. 10.1007/978-1-59745-241-0_17 19301678

[B37] Mylotte L. A. Duffy A. M. Murphy M. O’Brien T. Samali A. Barry F. (2008). Metabolic flexibility permits mesenchymal stem cell survival in an ischemic environment. *Stem Cells* 26 1325–1336. 10.1634/stemcells.2007-1072 18308942

[B38] Okuyama H. Krishnamachary B. Zhou Y. F. Nagasawa H. Bosch-Marce M. Semenza G. L. (2006). Expression of vascular endothelial growth factor receptor 1 in bone marrow-derived mesenchymal cells is dependent on hypoxia-inducible factor 1. *J. Biol. Chem.* 281 15554–15563. 10.1074/jbc.m602003200 16574650

[B39] Paquet J. Deschepper M. Moya A. Logeart-Avramoglou D. Boisson-Vidal C. Petite H. (2015). Oxygen tension regulates human mesenchymal stem cell paracrine functions. *Stem Cells Transl. Med.* 4 809–821. 10.5966/sctm.2014-0180 25979862 PMC4479617

[B40] Peach C. J. Mignone V. W. Arruda M. A. Alcobia D. C. Hill S. J. Kilpatrick L. E. (2018). Molecular pharmacology of VEGF-A isoforms: binding and signalling at VEGFR2. *Int. J. Mol. Sci.* 19:1264. 10.3390/ijms19041264 29690653 PMC5979509

[B41] Petit I. Jin D. Rafii S. (2007). The SDF-1-CXCR4 signaling pathway: a molecular hub modulating neo-angiogenesis. *Trends Immunol.* 28 299–307. 10.1016/j.it.2007.05.007 17560169 PMC2952492

[B42] Rios-Navarro C. Hueso L. Minana G. Nunez J. Ruiz-Sauri A. Sanz M. J. (2018). Coronary serum obtained after myocardial infarction induces angiogenesis and microvascular obstruction repair. Role of hypoxia-inducible factor-1A. *Rev. Esp. Cardiol.* 71 440–449. 10.1016/j.rec.2017.06.019 28751164

[B43] Rosova I. Dao M. Capoccia B. Link D. Nolta J. A. (2008). Hypoxic preconditioning results in increased motility and improved therapeutic potential of human mesenchymal stem cells. *Stem Cells* 26 2173–2182. 10.1634/stemcells.2007-1104 18511601 PMC3017477

[B44] Rubina K. Kalinina N. Efimenko A. Lopatina T. Melikhova V. Tsokolaeva Z. (2009). Adipose stromal cells stimulate angiogenesis via promoting progenitor cell differentiation, secretion of angiogenic factors, and enhancing vessel maturation. *Tissue Eng. Part A* 15 2039–2050. 10.1089/ten.tea.2008.0359 19368510

[B45] Ryou M. G. Mallet R. T. (2018). An in vitro oxygen-glucose deprivation model for studying ischemia-reperfusion injury of neuronal cells. *Methods Mol. Biol.* 1717 229–235. 10.1007/978-1-4939-7526-6_18 29468596

[B46] Shafiq M. Jung Y. Kim S. H. (2016). Insight on stem cell preconditioning and instructive biomaterials to enhance cell adhesion, retention, and engraftment for tissue repair. *Biomaterials* 90 85–115. 10.1016/j.biomaterials.2016.03.020 27016619

[B47] Stubbs S. L. Hsiao S. T. Peshavariya H. M. Lim S. Y. Dusting G. J. Dilley R. J. (2012). Hypoxic preconditioning enhances survival of human adipose-derived stem cells and conditions endothelial cells in vitro. *Stem Cells Dev.* 21 1887–1896. 10.1089/scd.2011.0289 22165914

[B48] Valorani M. G. Montelatici E. Germani A. Biddle A. D’Alessandro D. Strollo R. (2012). Pre-culturing human adipose tissue mesenchymal stem cells under hypoxia increases their adipogenic and osteogenic differentiation potentials. *Cell Prolif.* 45 225–238. 10.1111/j.1365-2184.2012.00817.x 22507457 PMC6622217

[B49] Vizoso F. J. Eiro N. Cid S. Schneider J. Perez-Fernandez R. (2017). Mesenchymal stem cell secretome: toward cell-free therapeutic strategies in regenerative medicine. *Int. J. Mol. Sci.* 18:1852. 10.3390/ijms18091852 28841158 PMC5618501

[B50] Wang Q. Xiao D. D. Yan H. Zhao Y. Fu S. Zhou J. (2017). The morphological regeneration and functional restoration of bladder defects by a novel scaffold and adipose-derived stem cells in a rat augmentation model. *Stem Cell Res. Ther.* 8:149. 10.1186/s13287-017-0597-z PMC5482942 28646909

[B51] Xu Y. Fu M. Li Z. Fan Z. Li X. Liu Y. (2016). A prosurvival and proangiogenic stem cell delivery system to promote ischemic limb regeneration. *Acta Biomater.* 31 99–113. 10.1016/j.actbio.2015.12.021 26689466 PMC6531039

[B52] Yue Y. Zhang P. Liu D. Yang J. F. Nie C. Yang D. (2013). Hypoxic preconditioning enhances the viability of ADSCs to increase the survival rate of ischemic skin flaps in rats. *Aesthetic Plast. Surg.* 37 159–170. 10.1007/s00266-012-9993-z 23232730

[B53] Zhang M. Xu M. X. Zhou Z. Zhang K. Zhou J. Zhao Y. (2014). The differentiation of human adipose-derived stem cells towards a urothelium-like phenotype in vitro and the dynamic temporal changes of related cytokines by both paracrine and autocrine signal regulation. *PLoS One* 9:e95583. 10.1371/journal.pone.0095583 24752317 PMC3994076

[B54] Zhe Z. Jun D. Yang Z. Mingxi X. Ke Z. Ming Z. (2016). Bladder acellular matrix grafts seeded with adipose-derived stem cells and incubated intraperitoneally promote the regeneration of bladder smooth muscle and nerve in a rat model of bladder augmentation. *Stem Cells Dev.* 25 405–414. 10.1089/scd.2015.0246 26863067

[B55] Zisa D. Shabbir A. Mastri M. Taylor T. Aleksic I. McDaniel M. (2011). Intramuscular VEGF activates an SDF1-dependent progenitor cell cascade and an SDF1-independent muscle paracrine cascade for cardiac repair. *Am. J. Physiol. Heart Circ. Physiol.* 301 H2422–H2432. 21963833 10.1152/ajpheart.00343.2011 PMC3233810

